# Large Tunable Spin-to-Charge Conversion in Ni_80_Fe_20_/Molybdenum Disulfide by Cu Insertion

**DOI:** 10.1021/acsami.4c03360

**Published:** 2024-04-26

**Authors:** Shu Hsuan. Su, Tzu Tai Huang, Bi-Rong Pan, Jung-Chuan Lee, Yi Jie Qiu, Pei-Yu Chuang, Pangihutan Gultom, Cheng-Maw Cheng, Yi-Chun Chen, Jung-Chung Andrew Huang

**Affiliations:** † Department of Physics, National Cheng Kung University, Tainan 701, Taiwan; ‡ Sheng Chuang Technology Company, Taichung 407330, Taiwan; § Department of Applied Physics, National University of Kaohsiung, Kaohsiung 811726, Taiwan; ∥ National Synchrotron Radiation Research Center, Hsinchu 300, Taiwan; ⊥ Department of Photonics, National Sun Yat-sen University, Kaohsiung 80424, Taiwan; # Taiwan Consortium of Emergent Crystalline Materials, Ministry of Science and Technology, Taipei 106, Taiwan

**Keywords:** spin-to-charge conversion, MoS_2_, inverse edelstein effect length, spin−orbital coupling

## Abstract

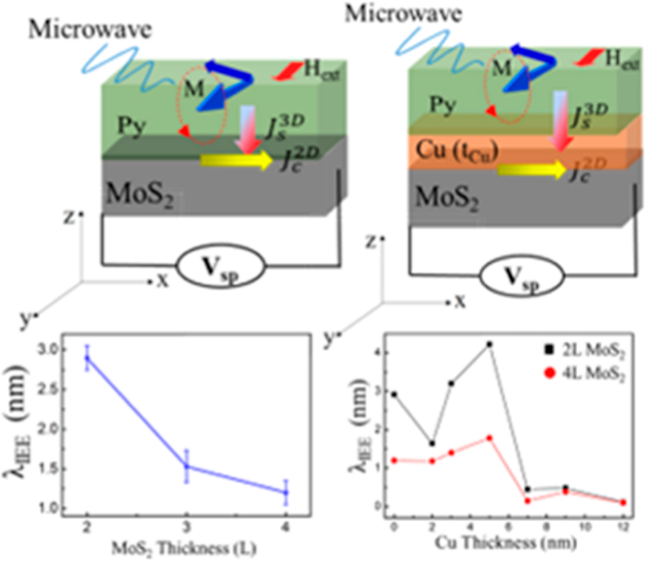

Spin-to-charge conversion at the interface between magnetic materials and transition metal dichalcogenides has drawn great interest in the research efforts to develop fast and ultralow power consumption devices for spintronic applications. Here, we report room temperature observations of spin-to-charge conversion arising from the interface of Ni_80_Fe_20_ (Py) and molybdenum disulfide (MoS_2_). This phenomenon can be characterized by the inverse Edelstein effect length (λ_IEE_), which is enhanced with decreasing MoS_2_ thicknesses, demonstrating the dominant role of spin–orbital coupling (SOC) in MoS_2_. The spin-to-charge conversion can be significantly improved by inserting a Cu interlayer between Py and MoS_2_, suggesting that the Cu interlayer can prevent magnetic proximity effect from the Py layer and protect the SOC on the MoS_2_ surface from exchange interactions with Py. Furthermore, the Cu–MoS_2_ interface can enhance the spin current and improve electronic transport. Our results suggest that tailoring the interface of magnetic heterostructures provides an alternative strategy for the development of spintronic devices to achieve higher spin-to-charge conversion efficiencies.

## 1 Introduction

The conversion of spin current into charge current and vice versa is a crucial phenomenon for integrating the spin and charge degree of freedom of electron behavior, with the goal of developing devices that utilize the information carried by electron spins. Until recently, the spin Hall effect (SHE) and its Onsager reciprocal, the inverse spin Hall effect (ISHE), were the only known mechanisms for bidirectional conversion.^1−3^ These effects rely on electron scattering processes in three-dimensional materials with significant SOC, including heavy metals (HM) like Pt, Pd, and Ta,^4−6^ antiferromagnetic materials such as IrMn_3_, Mn_2_Au and Mn_3_Sn,^7−9^ and semiconductors.^10−12^ Recently, several materials in the transition metal dichalcogenide (TMD) family had been studied to improve spin and charge conversion efficiency.^13,14^ Rashba-type SOC in TMDs can enable effective conversion from charge current to spin current by the direct Edelstein effect (EE)^15,16^ and vice versa through the inverse Edelstein effect (IEE).^17,18^ Among the various TMDs, molybdenum disulfide (MoS_2_) is one of the most stable materials, consisting of three covalently bonded hexagonal atomic layers (S–Mo–S) and capable of forming of two-dimensional (2D) layers. It is known that MoS_2_ has unique electronic properties and exhibits strong dependence on the thickness and mechanical strain.^19,20^ Recently, SOC-based approaches for spin injection, spin detection, spin transport, and spin-to-charge conversion in MoS_2_ have been attractive for spintronics.^17,21,22^ Liang et al. reported electrical spin injection and detection in the conduction band of a multilayer MoS_2_ channel using a two-terminal spin-valve configuration geometry and demonstrating spin-transport in MoS_2_ with relatively long spin-diffusion lengths larger than 200 nm.^21^ The current-induced spin-torque resonance in the Py/MoS_2_ bilayer indicates that MoS_2_ induces both field-like and damping-like torques, which further excite the ferromagnetic resonance (FMR) in Py.^23^ In addition, spin pumping in Co_2_FeAl (CFA)/MoS_2_ heterostructures has been reported.^24^ Large changes in effective damping parameter are associated with the orbital hybridization at the CFA/MoS_2_ interface, as confirmed by the density functional theory calculations of a CFA/MoS_2_ bilayer model. Furthermore, ferromagnets (FM)/MoS_2_ has generated interest in spin-charge conversion owing to the large SOC derived from the d orbitals of transition metals^25^ and the crystal structure lacks inversion symmetry.^26^ Spin-to-charge conversion efficiencies at room temperature (RT) have been reported in the Y_3_Fe_5_O_12_(YIG)/MoS_2_,^27^ Co_2_FeSi (CFS)/MoS_2_,^28^ and Py/MoS_2_^17^ systems.

To improve the efficiency of spin-injection via the ISHE, it has been proposed to use an intercalator such as Cu, Ag or NiO as a potential barrier between FM and HM.^29−31^ This can effectively tune the spin-dependent interfacial resistivity and improve spin injection. Cu has been widely used to control spin transmissivity in spintronic devices.^32^ The spatial mapping of spin accumulation in Cu due to the spin-pumping effect was observed using scanning transmission X-ray microscopy.^33^ Additionally, the Cu layer is used to eliminate proximity-induced ferromagnetism in FM for ISHE or IEE measurements.^34,35^ However, while Cu inserted layers have been employed in IEE measurements, their effectiveness in enhancing efficiency is not guaranteed. For example, in the NiFe/HgCdTe/HgTe system,^36^ P. Noel et al. observed a detrimental effect resulting from the direct contact of NiFe with HgTe, which was further deteriorated by the addition of a Cu interlayer. The produced charge current is considerably reduced in a NiFe/Cu/HgTe sample compared to that in NiFe/HgTe and a NiFe/HgCdTe/HgTe samples without Cu spacer layers. NiFe/Cu/HgTe samples exhibit a larger damping parameter and considerably smaller conversion efficiencies. Therefore, the spin-to-charge conversion efficiency is highly dependent on the choice of metal in contact, emphasizing the importance of careful selection the interlayer. Moreover, despite these recent advances, quantitative studies of the spin-to-charge conversion of FM/MoS_2_ are still lacking with respect to the effect of different MoS_2_ thicknesses and the effect of Cu intercalation.

In this study, we report a systematic study of the spin-to-charge conversion at RT in Py/MoS_2_ with different MoS_2_ thicknesses and Py/Cu/MoS_2_ with different Cu thicknesses (*t*_cu_). We found that λ_IEE_ increases with decreasing MoS_2_ thickness in Py/MoS_2_ due to the change in SOC magnitude. The dramatic improvement in λ_IEE_ by inserting the Cu layer into Py/MoS_2_ reveals that the Cu interlayer prevents the magnetic proximity effects from the Py layer and protects the SOC on the MoS_2_ surface from the exchange interaction with Py. Our results not only provide a correlation between the Cu interlayer and SOC strength, but also demonstrate the crucial role of optimizing the interface for spin-to-charge conversion.

## 2 Experiment Section

(0001)-oriented sapphire was chosen as a good van der Waals epitaxial substrate due to its atomically flat surface with no surface dangling bonds.^37^ Prior to sulfurization, Mo films of various thicknesses were deposited on sapphire substrates using an ion beam sputtering system. After metal deposition, the Mo films were placed in the center of a hot furnace for sulfurization. During the sulfurization process, nitrogen was used as a carrier gas while the furnace pressure was maintained at 0.7 Torr. Sulfur powder was used to sulfurize the Mo films at 800 °C to form MoS_2_ layers. The MoS_2_ samples were then transferred into a pulsed-laser deposition (PLD) chamber to grow Py, Cu, and Al layers at RT. In this work, the thickness of the Py and Al capping layers are fixed at 15 and 2 nm, respectively, to prevent environmental contamination.^35^

Structural and surface characterizations were performed using X-ray diffraction (XRD), X-ray reflectivity (XRR), high-resolution transmission electron microscope (HRTEM), and atomic force microscopy (AFM). Raman spectra were recorded on a micro-Raman spectrometer (Horiba-Jobin Yvon LabRAM-HR) with an excitation wavelength of 532 nm. X-ray photoemission spectroscopy (XPS) and angle-resolved photoemission spectroscopy (ARPES) were performed at beamline 24 A and BL21B1 of the Taiwan Light Source of the National Synchrotron Radiation Research Center.^38^ The Au 4f_7/2_ peak (84 eV) was used to calibrate the photon energy.

For FMR and spin-pumping measurements, we employed device structures comprising Al/Py/MoS_2_ and Al/Py/Cu/MoS_2_. Spin currents were generated in Py using FMR and injected into MoS_2_. For FMR measurement, microwaves were generated using a network analyzer (N5230A, Agilent Technologies).^35,39^ The Al/Py/MoS_2_ and Al/Py/Cu/MoS_2_ samples were facing down and attached to a coplanar waveguide (CPW). A GMW made electromagnet was used to apply an external magnetic field. The obtained data were fitted to sums of symmetric and antisymmetric Lorentzian functions. For the spin-pumping experiments, we excited the FMR by sweeping the external magnetic field while fixing the excitation microwave frequency (between 2.5 and 5 GHz) and measured the resulting DC voltage using a nanovoltmeter (Agilent 34420A). All spin-pumping measurements were performed at RT. The geometry of the samples we used to study spin pumping and resistivity is identical, with a sample width of ∼4 mm and a sample length of ∼7 mm. The sample resistivity was measured using a four-probe method.

## 3 Result and Discussions

**Figure 1:**
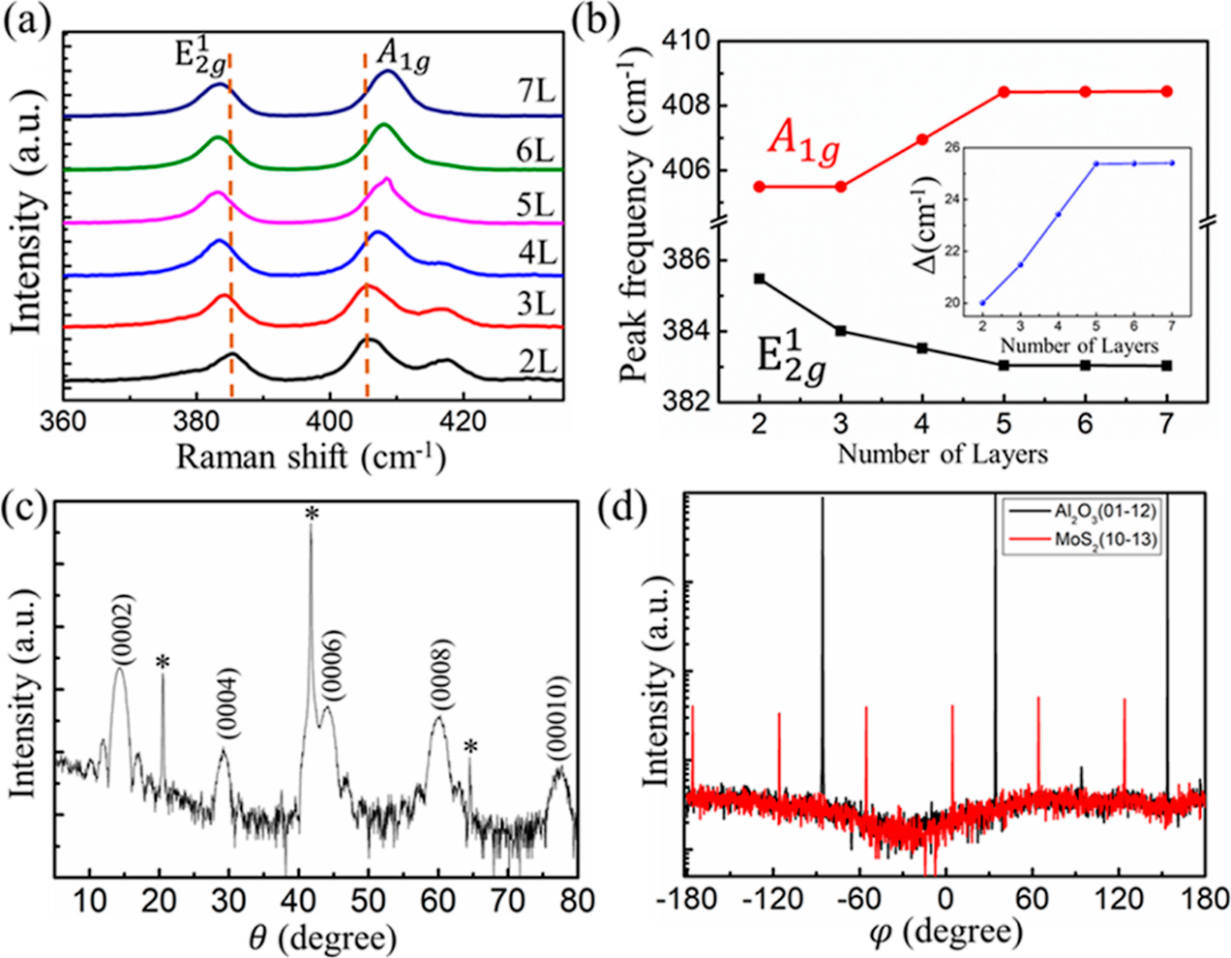
(a) Raman spectra of MoS_2_ films showing the E_2g_^1^ and A_1g_ modes. The distance between the E_2g_^1^ and A_1g_ peaks (indicated by the dashed lines) is ∼20.6 cm^–1^, confirming the 2L thickness of this particular MoS_2_ film. The additional Raman peak at ∼418 cm^–1^ is from the sapphire substrate. (b) The two characteristic Raman peaks E_2g_^1^ and A_1g_ as a function of layer thickness. Inset: The peak frequency difference (Δ) can be used to determine the number of MoS_2_ layers. (c) XRD pattern of MoS_2_ on *c*-plane sapphire substrate (indicated by *). (d) ϕ-Scan at MoS_2_ (101̅3) diffraction position and sapphire (011̅2)position.

The controllability of the number of layers is an essential issue for the growth of 2D crystal. Under the condition of sufficient sulfur, the number of layers of MoS_2_ should be tuned according to the thickness of the Mo metal film. It is generally accepted that the frequency shift of the Raman peak can determine the number of MoS_2_ layers.^40,41^ The MoS_2_ thin films were characterized using Raman spectroscopy, as shown in Figure 1a. Two characteristic Raman peaks E_2g_^1^ and A_1g_ are observed for all samples, representing the in-plane and out-of-plane vibrational modes of the MoS_2_ films. The two specific MoS_2_ peaks are located at ∼385 and ∼403 cm^–1^, respectively. As the number of layers increases, the van der Waals forces of the MoS_2_ interlayer suppress the atomic vibrations, resulting in higher force constants.^42^ Thus, both the E_2g_^1^ and A_1g_ modes should be stiffened. The observed blue shift of the A_1g_ peak is consistent with the predicted stiffening as the number of layers increases. In contrast, the E_2g_^1^ peak shows a red-shift instead of a blue-shift, suggesting that the increase of the interlayer van der Waals forces play a minor role in the stacking induced structural changes or in the long-range Coulomb interlayer interactions in multilayer of MoS_2_,^43^ which may dominate the change in atomic vibrations. For MoS_2_ films with five or more layers, the frequencies of both modes converge to the bulk values. Note that the opposite variation of these two Raman peaks allows the use of their frequency difference (Δ) to identify the number of MoS_2_ layers, as shown in the inset of Figure 1b. These results are in good agreement with the observations of exfoliated MoS_2_ layers.^41^ The MoS_2_ thickness was also inferred using XRR measurements in Figure S1. The crystalline quality of the MoS_2_ thin films was analyzed by XRD measurements. The out-of-plane θ–2θ scans of six-layer MoS_2_ reveal the (0001) family diffractions of MoS_2_, indicating the preferred growth orientation of MoS_2_ with its *c*-axis parallel to the Al_2_O_3_ (0001) substrate, as shown in Figure 1c. The azimuthal ϕ scan of the (101̅3) plane of the MoS_2_ films and the (011̅2) plane of the Al_2_O_3_ substrate are shown in Figure 1d, where ϕ is the azimuthal angle of the sample relative to the sample normal. Due to the 6-fold symmetry of the hexagonal MoS_2_ phase, the full range of the φ = 360° scan shows six peaks at the MoS_2_ (101̅3) position. A full range ϕ = 360° scan of the Al_2_O_3_ (011̅2) substrate at the same region revealed three peaks of single-crystalline sapphire corresponding to its triple symmetry. The ϕ-scan results confirm that the unit cell of MoS_2_ is rotated by 30° relative to the unit cell of the Al_2_O_3_ substrate.^44^ HRTEM measurements show ordered crystalline MoS_2_ in Figure S2, confirming the XRD results and indicating that the growth direction of MoS_2_ is along the *c*-axis of the sapphire substrate and the MoS_2_ film has a uniform periodic arrangement of atoms throughout the region. The AFM topography of the MoS_2_ layers shown in Figure S4 exhibits a surface root-mean-square (rms) roughness of about 0.47 nm, indicating the large-area smooth surface. The rms roughness value we obtained for MoS_2_ is in line with the findings of other research groups.^17^

**Figure 2:**
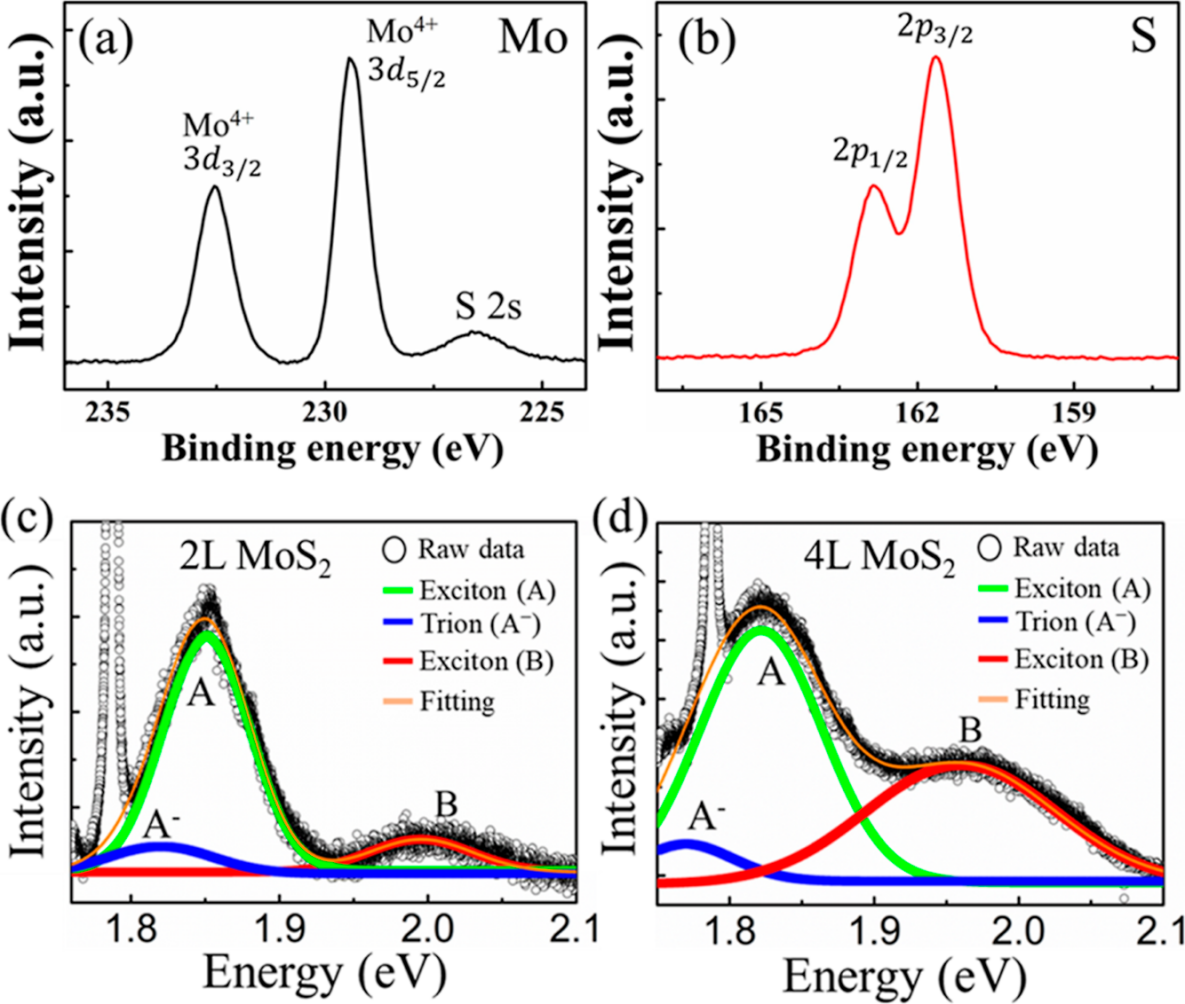
XPS spectra of (a) Mo 3d and S 2s peaks and (b) S 2p peaks. PL spectra for (c) 2L and (d) 4L MoS_2_ samples. Experimental data were fitted using three Gaussian functions corresponding to A and B excitons and negative trion (A^–^) peaks. The solid green, red and blue lines represent the fits for exciton A, B, and trion (A^–^), respectively. The solid orange line is the cumulative fit of all three components.

XPS was used to investigate the chemical nature of the MoS_2_ samples. Figure 2a,b show the binding energies of Mo and S in the 6L MoS_2_ thin film. Peaks representing Mo^4+^ 3d and S^2–^ 2p orbitals were observed. Due to the spin–orbit interaction, the Mo^4+^ 3d orbital splits into Mo^4+^ 3d_5/2_ and Mo^4+^ 3d_3/2_ orbitals with binding energies of 229.8 and 233 eV, respectively. Similarly, the S^2–^ 2p orbital splits into S^2–^ 2p_3/2_ and S^2–^ 2p_1/2_ orbitals with binding energies of 162.5 and 163.5 eV, respectively. These results are in agreement with the reported values.^45^ The XPS studies suggest that all Mo metals were converted to MoS_2_. Moreover, the band structure of 7L MoS_2_ thin films were observed by ARPES, showing indirect band gap behavior in Figure S6. We also performed PL measurements on 2L and 4L MoS_2_, as illustrated in Figures 2c,d, respectively. These PL spectra were fitted with three Gaussian functions, including trion (A^–^), A-exciton, and B-exciton. Due to the presence of extra electrons bound to the excitons, the trion component can be observed as part of the strongest band.^46^ The peaks of the A and B exciton have been identified as direct exciton transitions at the Γ point of the Brillouin zone^47^ Their energy difference is caused by the splitting of the valence band due to the presence of SOC. The spin–orbit splitting energies of 2L and 4L MoS_2_ are 139 and 129 meV, respectively. This indicates that the SOC in 2L MoS_2_ is relatively strong, with increasing MoS_2_ thickness, the intrinsic spin–orbit coupling decreases, which is consistent with theoretical calculations.^48^

**Figure 3:**
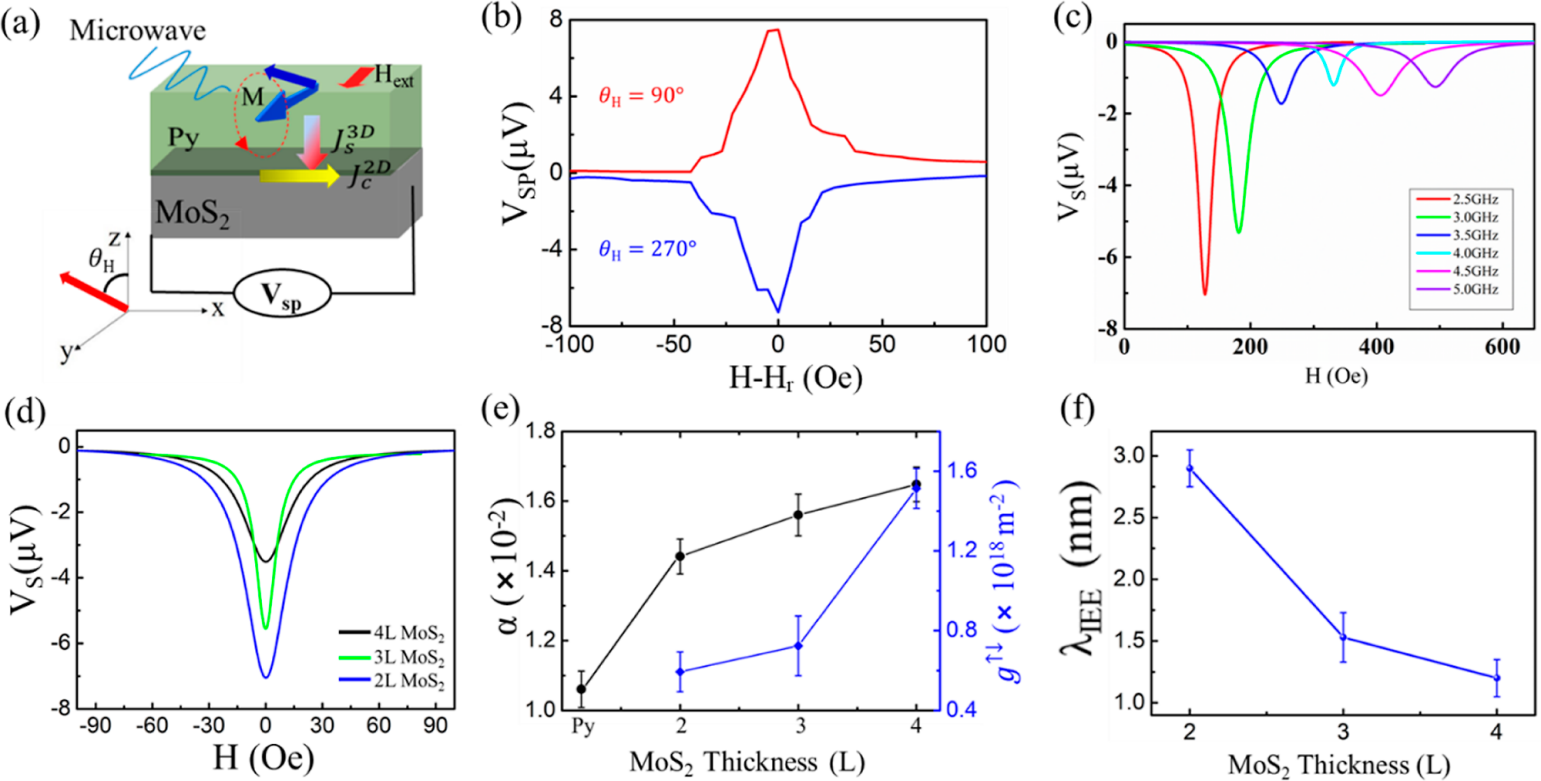
(a) Schematic diagram of the experimental setup for spin-pumping measurement on Py/MoS_2_ samples. (b) Spin-pumping voltage in Py/2L MoS_2_ sample vs *H* – *H*_r_ spectra at θ_H_ = 90 and 270° (two opposite in-plane fields) at a microwave frequency of 2.5 GHz and a microwave power of 32 mW. (c) The extracted *V*_s_ voltage in Py/2L MoS_2_ measured at various excitation frequencies. (d) The extracted *V*_s_ voltage in Py/MoS_2_ samples for three different MoS_2_ thicknesses. (e) Spin-mixing conductance *g*^↑↓^ (right axis) and magnetic damping constant α (left axis) as a function of MoS_2_ thickness. (f) A summary of the λ_IEE_ of the Py/MoS_2_ as a function of MoS_2_ thickness.

A representative schematic diagram of the device structure used for FMR, and spin-pumping measurements is shown in Figure 3a. To verify the quality of MoS_2_ after Py layer deposition, Raman spectroscopy of Py/MoS_2_ was performed in Figure S7. The peak positions are consistent with the corresponding pristine MoS_2_, indicating that the quality of MoS_2_ is not affected after the Py layers deposition. Furthermore, the AFM image of Py/MoS_2_ bilayer (in Figure S5) shows a flat surface with a rms roughness of about 0.347 nm. The interfacial quality of the Py/MoS_2_ bilayer was examined by HRTEM in Figure S3. The interface between Py and MoS_2_ is flat and continuous, which is important for efficient spin pumping.

The microwaves produced by an RF generator transmitted through the CPW, which causes the magnetization of the Py layer to precession at GHz frequencies. Under a certain resonance field, when the frequency of the magnetic field matches the oscillation frequency of the Py layer, the spin current generated from the Py layer injects into the MoS_2_ layer due to the spin pumping effect.^17^ The injected spin current is then converted to a DC charge current caused by the interfacial inverse Edelstein^22^ and the bulk inverse spin Hall effects.^49^ The charge current can be determined by measuring the spin-pumping induced voltage *V*_SP_ with a nanovoltmeter.

We fit the data the symmetric and antisymmetric components using the following Lorentz equation^35,39^
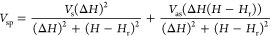
1where *V*_s_ and *V*_as_ are the symmetric and antisymmetric components of the measured voltage. *H*_r_ is the resonance field and Δ*H* is the peak-to-peak line width obtained from the microwave absorption derivative d*P*/d*H*. In fitting eq 1, we evaluate *V*_s_ and *V*_as_; the coefficients of the symmetric component (*V*_s_) consist of the spin-to-charge voltage caused by the IEE or ISHE signal generated by spin pumping; the antisymmetric part (*V*_as_) comes from the anomalous Hall effect (*V*_AHE_) or anomalous magnetoresistance (*V*_AMR_). A detailed analysis is provided in Figure S9. Figure 3b shows spin pumping voltage obtained by sweeping the magnetic-field (*H*), measured in the Py/MoS_2_ sample for two in-plane field directions, namely θ = 90 and 180°. The observed spin pumping signal changes sign as expected from the IEE, which arises from the spin accumulation induced by the Rashba spin–orbit interaction. Figure 3c depicts the magnetic field dependence of the extracted *V*_s_ obtained from the Py/2L MoS_2_ sample at various microwave frequencies. The obtained *V*_s_ decreases with increasing frequency, resulting from variation of the performance of the microwave transmission line at different frequencies, which is consistent with the previous report.^50,51^ The decrease in spin pumping voltage with increasing frequency can be elucidated through several mechanisms: (i) at higher frequencies, the magnetization precession occurs more rapidly. However, the efficiency of conversion of this precession into a spin current that can be injected into the adjacent layer might decrease due to mismatches in the dynamic susceptibility of the ferromagnet and the spin-mixing conductance at the interface. (ii) With increasing frequencies, the electromagnetic skin depth decreases, resulting in reduced penetration of electromagnetic waves (and hence the spin waves associated with the magnetization precession) into the material, consequently lowering the total generated spin current. (iii) The interaction between the ferromagnet and the adjacent nonmagnetic material can exhibit resonant effects at certain frequencies. The higher frequencies induce the off-resonance, potentially reducing spin pumping efficiency. (iv) The damping parameter is frequency-dependent; at higher frequencies, the intrinsic and extrinsic mechanisms contributing to damping (such as magnon–magnon and magnon-phonon scattering) may become more significant, leading to a reduction in the efficiency of spin pumping.

At ∼ 2.5 GHz, the observed signal is larger than at the other frequencies. Therefore, the frequency field for the spin-to-charge conversion efficiency analysis was determined to be 2.5 GHz. Figure 3d compares the extracted *V*_s_ of the 2L to 4L MoS_2_ samples, revealing a notably stronger signal for 2L MoS_2_ compared to other thicknesses. The relationship between frequency *f* and *H*_r_ was obtained for samples with different MoS_2_ thicknesses in Figure S8. The data conform to the Kittel’s formula, , where γ is the gyromagnetic ratio to extract the effective saturation magnetization (*M*_eff_). Δ*H* is plotted as a function of excitation frequency for MoS_2_ samples in Figure S8. The damping constant (α) is obtained by fitting Δ*H* to *f* with this formula, , where *H*_0_ corresponds to the presence in the Py layer. α as a function of MoS_2_ samples with various thickness is presented in Figure 3e (left *y*-axis). The α enhancement of the Py/MoS_2_ samples compared to the single Py sample indicates the presence of spin injection via spin pumping^17,22^ and possibly other effects such as magnetic proximity^52^ and spin memory loss.^53^ The difference in the damping constants gives the spin injection efficiency, known as the spin mixing conductivity(*g*^↑↓^) and represents the global spin transmission. The spin-mixing conductivity is determined by^35,39^
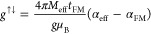
2where *g*, μ_B_,*t*_FM_,α_eff_ and α_FM_ are the Landé *g*-factor, Bohr magneton, Py layer thickness, and damping constant of MoS_2_ samples and pure Py, respectively. The *g*^↑↓^ obtained from eq 2 as a function of MoS_2_ thickness is shown on the right axis of Figure 3d. The obtained values of *g*^↑↓^ are lower than those previously reported values for Py/MoS_2_,^22^ and comparable to those of the YIG/MoS_2_^27^ and Co_40_Fe_40_B_20_/MoS_2_ system.^54^

To obtain the IEE length from the experimental data, we performed a standard spin-pumping mode analysis to obtain the injected vertical spin-current density (*J*_s_^3D^) generated by the magnetization precession in the ferromagnetic Py layer. The general expression for *J*_s_^3D^ is given by eq 3.
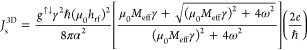
3in which *M*_eff_, ω, *g*^↑↓^, *h*_rf_, ℏ and *e* are the effective saturation magnetization, excitation frequency, spin-mixing conductivity, microwave RF field, Planck constant and electronic charge, respectively. *h*_rf_ is the RF field generated due to the RF current of frequency *f* = ω/2π flowing through the CPW.

After spin pumping into the MoS_2_ layer, a charge current is generated in the MoS_2_ layer and detected as a potential drop *V*_sp_ across the measured sample.^34^ Therefore, the charge current density *J*_C_^2*D*^ that is generated by the *J*_s_^3D^ pumping, can be expressed with eq 4.^35,39^
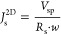
4where *w* and *R*_s_ are the device width and the sample resistance, respectively. The efficiency of the spin-to-charge conversion is given by .^35,39^Figure 3f shows λ_IEE_ as a function of MoS_2_ thickness. The λ_IEE_ displays the thickness dependence. For the 2L MoS_2_ case, λ_IEE_ shows a maximum value, λ_IEE_ = 2.9 nm, with increasing MoS_2_ thickness, the value of λ_IEE_ decreases. The decay in efficiency with increasing MoS_2_ layer thickness indicates that the spin-to-charge conversion is dominated by interfacial IEE, rather than bulk ISHE.^51^ The trend is consistent with observations in the other topological insulator systems.^51,55^

The PL measurements in Figures 2c,d also shows the SOC magnitude in 4L MoS_2_ is smaller than in 2L MoS_2_, which may reduce the spin-to-charge efficiency in 4L MoS_2_. The SOC dependent IEE lengths were also observed in other TMD systems.^17^ In addition, the λ_IEE_ values in this result are much greater than the previously reported, 0.01 nm in CFS/MoS_2_,^28^ 0.4 nm in YIG/MoS_2_^27^ and 0.54 nm in Py/1L MoS_2_.^17^ It is important to note that the magnitude of the IEE coefficient is expected to critically depend on the interfacial properties between the MoS_2_ and FM layer. We speculate that the sputtering techniques used for FM layer deposition in most other studies, characterized by higher bombardment energies and low vacuum conditions, could inevitably damage the FM/MoS_2_ interface during growth, significantly impacting the value of λ_IEE_. In contrast, our FM layer (Py) was deposited using the PLD method, which employs lower bombardment energies and operates under high vacuum conditions, potentially reducing contamination and interfacial damage. Furthermore, employing different FM layers as spin injection layers may lead to variations in interfacial spin polarization due to the hybridization effect at the FM/MoS_2_ interface.^56,57^ Additionally, varying MoS_2_ thickness also affects the value of IEE. Therefore, refining the composition, structure, and interface of the Py/MoS_2_ heterostructure in this work can help improve the efficiency of spin-to-charge conversion.

Furthermore, the magnitudes of λ_IEE_ in our system exceed the values found at Bi/Ag interfaces of 0.1–0.4 nm^58^ as well as other Rashba interfaces,^59^ however, they are comparable to the values observed in topological states, such as those in HgCdTe/HgTe (∼2.1 nm)^36^ and α-Sn (∼2.1 nm)^60^ systems.

**Figure 4:**
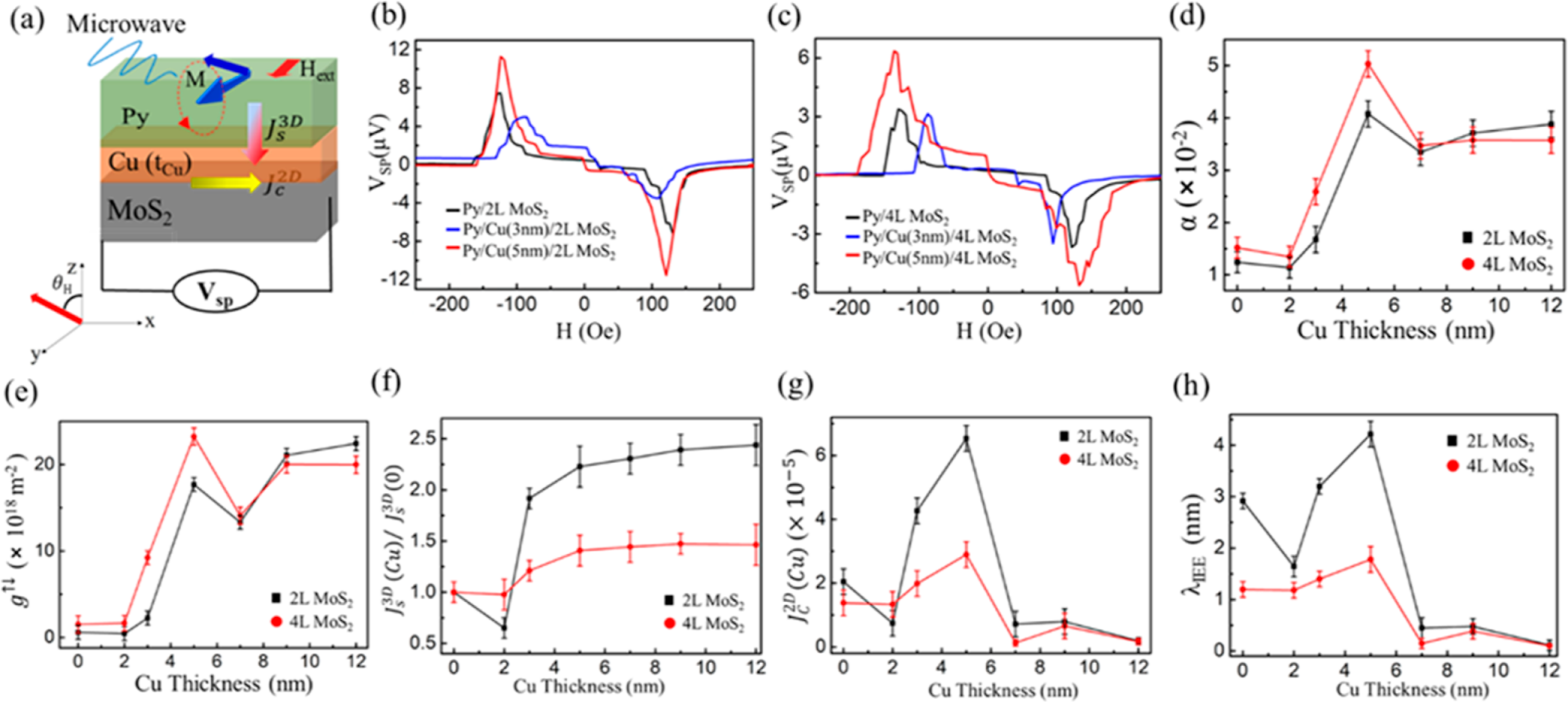
Spin pumping results for Py/Cu/2L MoS_2_ and Py/Cu/2L MoS_2_ with various thicknesses of the intercalation layer. (a) Schematic diagram of the experimental setup for spin-pumping measurement. The magnetic field-dependent on the voltage in (b) Py/Cu/2L MoS_2_ and (c) Py/Cu/4L MoS_2_ as a function of Cu thickness at a microwave frequency of 2.5 GHz and a microwave power of 32 mW. (d) The magnetic damping constant α_eff_ as a function of the Cu thickness. (e) The spin-mixing conductance *g*^↑↓^ as a function of Cu thickness. (f) Spin current normalized as a function of Cu thickness. (g) Charge-current density *J*_s_^2D^(Cu) as a function of the Cu thickness. (h) IEE length λ_IEE_ as a function of Cu-intercalated thickness.

However, the magnetism in FM/MoS_2_ can be induced by the magnetic proximity effect of adjacent magnetic layers and the interfacial hybridization at the interface,^61^ which will affect the spin-charge conversion. Several groups have attempted to separate the FM/SOC interface by introducing an intermediate layer such as Cu, which has long spin diffusion length.^29^ In this work, the spin pumping measurements with different Cu interlayer thicknesses inserted between Py and MoS_2_ is shown in Figure 4a. Figures 4b,c show the *V*_sp_ of Py/Cu(*t*_cu_)/2L MoS_2_ and Py/Cu(*t*_cu_)/4L MoS_2_ as a function of Cu insertion layer thickness, respectively. The magnitude of *V*_sp_ in Py/Cu/MoS_2_ sample is larger than that of Py/MoS_2_ sample, while a decay of *V*_sp_ is observed with increasing Cu thickness. Figure 4d shows the observed damping constant (α) as a function of the thickness of the Cu intercalation layer. In the case of 2L MoS_2_, the damping parameter increases from ∼1.2 (*t*_cu_ = 0 nm) to 4.1 (*t*_cu_ = 5 nm) when the Cu layers are inserted. The increase of the damping constant in the heterostructure can be understood as the generated spin current carrying angular momentum from Py into the nonmagnetic interlayer (Cu), which is lost to the lattice through spin-flips or diffuses further into the final spin sink. Due to conservation of angular momentum, a torque is generated that reduces the precession angle and thus increases the damping in Py. The trend of variation in the damping parameter of Py/Cu/4L MoS_2_ is similar to that of Py/Cu/2L MoS_2_. It should be noted that the damping parameter decreases as the thickness of the Cu intercalation layer increases in the Py/Cu/2L MoS_2_ and Py/Cu/4L MoS_2_ systems. Some possible explanations may elucidate this phenomenon: (i) the decrease in the antidamping parameter could be stem from the spin back-flow resulting from interfacial nonequilibrium spin accumulation. The theoretical model proposed by Y. Tserkovnyak et al.^32^ describes the generation of nonequilibrium spin density due to diffusive spin accumulation from spin pumping. According to this model, a pure transverse spin current (and hence spin angular momentum) flows out of the Py layer and accumulates in the adjacent normal metal (Cu) layer. Consequently, the nonequilibrium spin density created near the interface induces a back-flow of spin angular momentum (and thus pure spin current) into the Py layer, which suppresses the effective damping constant. (ii) Additionally, the presence of an Al capping layer might lead to the emergence of antidamping behavior.^62^ This effect can often be attributed to the formation of a protective layer of aluminum oxide on the surface of the Py layer, a result of passivation when exposed to air under normal atmospheric conditions. It is probable that this Al_2_O_3_ layer introduces structural inversion asymmetry (SIA) atop the Py layer. Similar generation of SIA on FM layer has also been observed by various research groups.^63,64^ The existence of SIA is known to facilitate nonequilibrium spin accumulation to undergo an inverse Rashba–Edelstein effect, which, in turn, generates a charge current supported by the presence of interfacial states near the interface.^58^ The resulting charge current could induce an antidamping spin–orbit torque (SOT) on the magnetization of the FM layer.^65^

Figure 4e shows the dramatic change of spin-mixing conductivity with the thickness of Cu intercalation layer. When varying *t*_cu_ from 5 to 12 nm, we obtained spin-mixing conductivity values in the range of 13–23 × 10^18^ m^–2^, which are comparable to those of CFA/MoS_2_^24^ and Bi/Ag Rashba interfaces.^58^ Moreover, we observed a strong nonmonotonic behavior of spin-mixing conductivity with increasing Cu thickness, which may be attributed to the oscillatory behavior caused by the quantum well state in the metal insertion layer^66^ and the magnetic anisotropy induced by the interlayer coupling in Py/Cu.^67^ Interestingly, λ_IEE_ does not follow a similar trend. Figure 4h shows the Cu thickness dependence of λ_IEE_. For the 2L MoS_2_ system, the value of λ_IEE_ drops significantly at *t*_cu_ = 2 nm. When *t*_cu_ is further increased to 5 nm, λ_IEE_ increases significantly to ∼4.1 nm, which is about 41% higher than the value for Py/2L MoS_2_. For the 4L MoS_2_ system, the maximum value of λ_IEE_ at *t*_cu_ = 5 nm is 1.7 nm, which is about 49% higher than that of Py/4L MoS_2_. For both Py/Cu/2L MoS_2_ and Py/Cu/4L MoS_2_ systems, a decay of λ_IEE_ was observed with a further increase in the thickness of the Cu interlayer.

To explain the observed phenomena and to reveal the roles of the Py/MoS_2_ interface and the Cu intercalated layer in the spin-to-charge conversion, the effect of the Cu interlayers on the *J*_s_^3D^ and *J*_C_^2D^ values need to be investigated. Figure 4f shows the *t*_cu_ dependence of *J*_s_^3D^(Cu), normalized by *J*_s_^3D^(0) which is the spin current detected in the Py/MoS_2_ without the intercalation of Cu. The normalized *J*_s_^3D^(Cu)/*J*_s_^3D^(0) initially decreases at *t*_cu_ = 2 nm, then increases with increasing Cu thickness, and eventually reaches a plateau. The initial decrease in *J*_s_^3D^(Cu)/*J*_s_^3D^(0) at *t*_cu_ = 2 nm may be related to the much higher resistivity of the thin Cu layer due to the finite size effect, which can lead to significant spin flipping.^32^ Spin accumulation in the Cu interlayer occurs when *t*_cu_ ≥ 3 nm.^33^ With increasing the Cu thickness, the trend of *J*_s_^3D^(Cu)/*J*_s_^3D^(0) confirms that the intercalated Cu layer indeed acts as a well spin transmitter with minimal spin dissipation. The dependence of *J*_s_^2D^(Cu) on *t*_cu_ is summarized in Figure 4g. The Cu intercalation layer from 3 to 5 nm allows higher *J*_s_^2D^(Cu) than that of direct contact between Py and MoS_2_, which is sufficient for the Cu layer to form a complete spacer between Py and MoS_2_, and the resistance of the Cu interlayer is significantly reduced.^29^ It is worth noting that the interface between Cu and MoS_2_ can facilitate a unique plasmon resonance at an energy of 1 eV, leading to enhanced electronic activity.^68,69^ Nevertheless, *J*_C_^2D^(Cu) decreases with increasing *t*_cu_ beyond 5 nm, suggesting that the positive spin Hall angle of the Cu interlayer generates a positive voltage response that reduce the overall response. Therefore, a thicker Cu intercalation layer (*t*_cu_ ≥ 5 nm) leads to a decrease in *J*_C_^2D^(Cu). The enhanced λ_IEE_ suggests that the overall spin-to-charge conversion in the Py/Cu/MoS_2_ trilayers is higher compared to the direct contact between Py and MoS_2_. This highlights the importance of optimizing the quality and thickness of the Cu interlayer and Cu–MoS_2_ interface to enhance the transport of spin current and electronic transport. Additionally, the use of the Cu layer serves to protect the SOC of the MoS_2_ surface from exchange interactions with Py, further enhancing the spin-to-charge conversion.

## 4 Conclusion

In summary, we have performed a comparative analysis of the spin-to-charge conversion efficiency in Py/MoS_2_ bilayers with varying thicknesses of MoS_2_ and Py/Cu/MoS_2_ trilayers with different Cu thicknesses at RT. Our findings demonstrate that the spin-to-charge conversion improves with decreasing MoS_2_ thickness in Py/MoS_2_ bilayers, highlighting the dominant role of SOC in MoS_2_. Additionally, we made a significant discovery that the inclusion of a suitable Cu interlayer substantially enhances the spin-to-charge conversion, suggesting that the Cu interlayer serves to mitigate the magnetic proximity effect from the Py layer and protect the SOC on the MoS_2_ surface. Moreover, the Cu–MoS_2_ interface can enhance the spin current and improve electronic transport. The results indicate that modification of the interface between MoS_2_ and the magnetic layer by Cu can be an alternative strategy to achieve higher spin-to-charge conversion efficiency for the advancement of low-power-consumption spintronic devices.

## Supporting Information Available

X-ray reflectometry (XRR) measurements of the MoS_2_ thin films, HRTEM study of the MoS_2_ and Al/Py/MoS_2_ structures, AFM images of the MoS_2_ and Py/MoS_2_ surface structures, ARPES measurements of the MoS_2_ thin films, Raman measurements of the MoS_2_ thin films before and after the deposition of Py and Al, Ferromagnetic dependence of peak-to-peak line width and resonance field, Numerical fitting procedure of obtained spin-pumping voltage (PDF)
