# Supplementary material for: Large Tunable Spin-to-Charge Conversion in Ni80Fe20/Molybdenum Disulfide by Cu Insertion
Source: ACS Appl Mater Interfaces. 2024 Apr 26;16(18):24122–31. doi: 10.1021/acsami.4c03360 (PMC11082844; doi:10.1021/acsami.4c03360)
Supplement: Supplementary file 1 — am4c03360_si_001.pdf [file am4c03360_si_001.pdf]

Supporting Information

Large Tunable Spin-to-Charge Conversion in $Ni_{80}Fe_{20}/MoS_2$ by Cu Insertion

Shu. Hsuan. Su[1,§], Tzu Tai Huang[1,§], Bi-Rong Pan[1], Jung-Chuan Lee[1,2], Yi Jie Qiu[1], Pei-Yu Chuang[4], Pangihutan Gultom[1], Cheng-Maw Cheng [4,5]*, Yi-Chun Chen[1], Jung-Chung Andrew Huang[1,3,6]**

[1]*Department of Physics, National Cheng Kung University, Tainan, Taiwan 701, Taiwan.*

[2] *Sheng Chuang Technology Company, Taichung 407330, Taiwan.*

[3] *Department of Applied Physics, National University of Kaohsiung, Kaohsiung 811726, Taiwan.*

[4] *National Synchrotron Radiation Research Center, Hsinchu 300, Taiwan.*

[5] *Department of Photonics, National Sun Yat-sen University, Kaohsiung 80424, Taiwan.*

[6]*Taiwan Consortium of Emergent Crystalline Materials, Ministry of Science and Technology, Taipei 106, Taiwan.*

[§]S. H. S. and T. T. H. contributed equally to this work.

[*]Address correspondence to J.-C.-A. Huang, jcahuang@mail.ncku.edu.tw S. H. Su, macg0510@yahoo.com.tw C.-M. Cheng, makalu@nsrrc.org.tw

Supplementary Note 1. X-ray reflectometry (XRR) measurements of the MoS_2 thin films

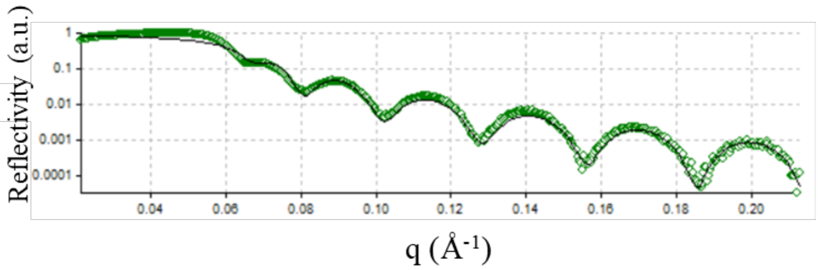

Figure S1. XRR pattern of the MoS_2 thin films.

XRR measurements provided insight into the properties of the MoS_2 thin films. The oscillation pattern indicated a sharp interface between the substrate and MoS_2. [1, 2] The fitting of XRR curve gave information about the thickness of the MoS_2 layer. The MoS_2 thickness in Figure S1 was estimated to be six layers.

Supplementary Note 2. HRTEM study of the MoS_2 and Al/Py/MoS_2 structures.

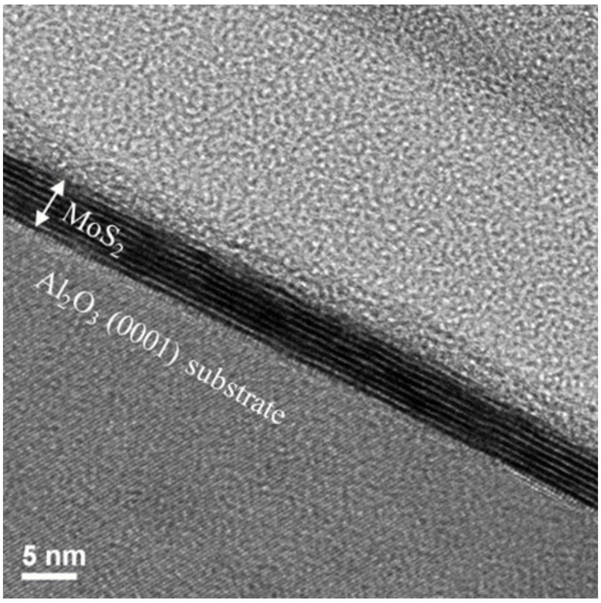

Figure S2. The cross-sectional TEM image of 6 L MoS_2 grown on *c*-plane sapphire substrate.

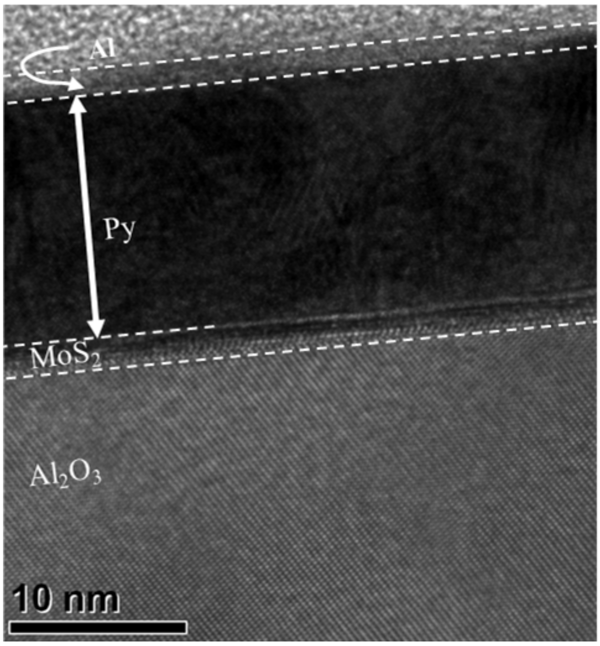

Figure S3. HRTEM image of the Al/Py/MoS_2 structure for spin pumping measurement.

The HRTEM image reveals cross-sectional geometry structure of the Al/Py/MoS_2 sample in Figure S3. The HRTEM image also confirms the existence of a clear and continuous interface between MoS_2 and Py, which is essential for efficient spin-pumping measurements.

Supplementary Note 3. AFM images of the MoS_2 and Py/MoS_2 surface structures.

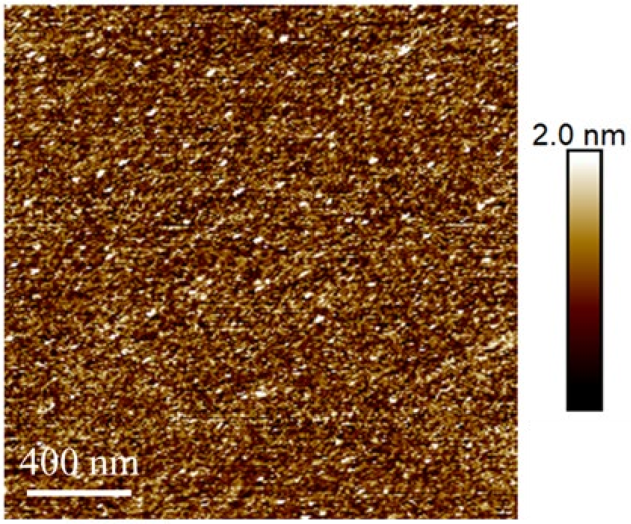

Figure S4. AFM image of the 2L MoS_2 grown on a *c*-plane sapphire substrate.

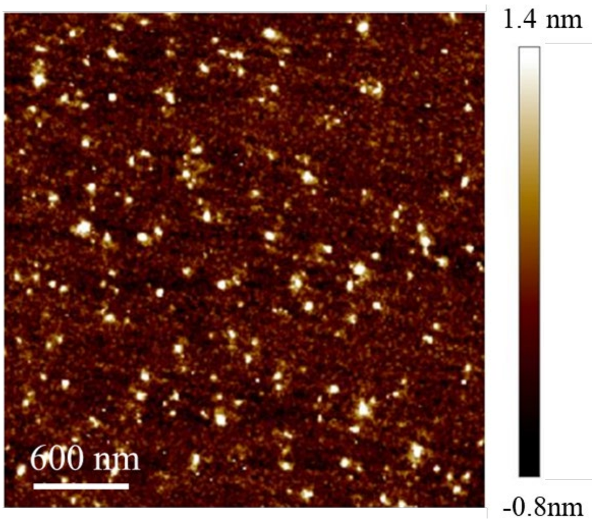

Figure S5. AFM image of the Py/2L MoS_2 bilayer.

Supplementary Note 4. ARPES measurements of the MoS_2 thin films.

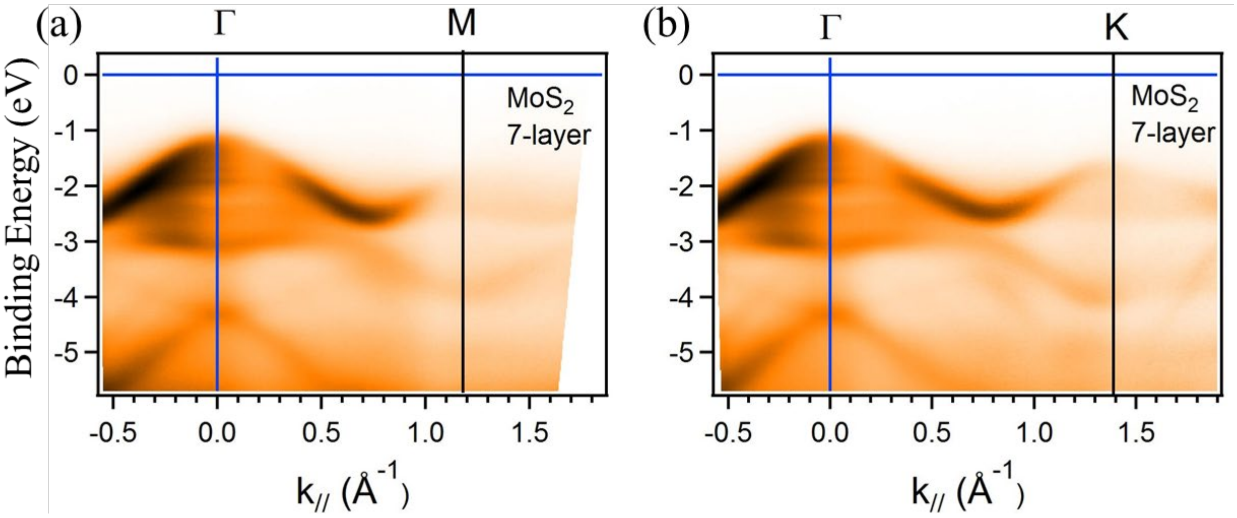

Figure S6. ARPES spectra of the as-grown 7L MoS_2 measured along directions (a) M-Γ-M and (b) K-Γ-K.

To study the band structure of MoS_2 thin films using ARPES measurements, it is necessary to transfer the thin films onto Si substrates. Figure S6 shows the band structures recorded along the Γ-M and Γ-K directions of the 7L MoS_2 thin films. The measured valence bands of MoS_2 comes from hybridization of the Mo 4*d* and S 3*p* orbitals. [3, 4] The band structures along the Γ-M and Γ-K directions are highly similar. The spacing between the valence band maximum (VBM) and the Fermi level (E_F) is about 1 eV, indicating that our 7L MoS2 sample is heavily electron-doped. Notably, the VBM at K is lower than that at Γ, indicating an indirect bandgap behavior.

Supplementary Note 5. Raman measurements of the MoS_2 thin films before and after the deposition of Py and Al

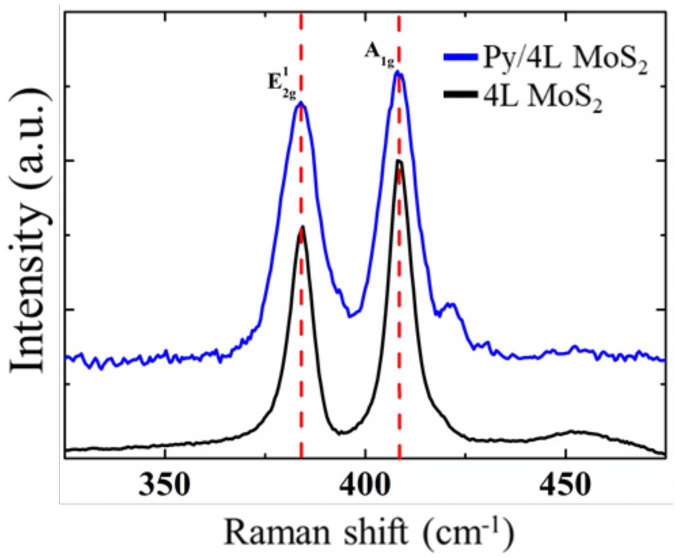

Figure S7. Raman spectra for 4L MoS_2 before and after the deposition of Py layers.

Figure S7 shows the Raman spectra of pristine 4L MoS_2 (black line), and after Al/Py bilayer deposition (blue line), respectively. For MoS_2, two peaks corresponding to E_{2g}^{1} and A_{1g} Raman modes were observed. The peak positions of the Raman spectra obtained after the deposition of Py and Al layers coincide with their corresponding pristine MoS_2. This demonstrates that the quality of the MoS_2 was not affected after the deposition of Py and Al layers. This reveals that there is no diffusion between Py and MoS_2, which would adversely alter the Raman signal from MoS_2.

Supplementary Note 6. Ferromagnetic dependence of peak-to-peak linewidth and resonance field

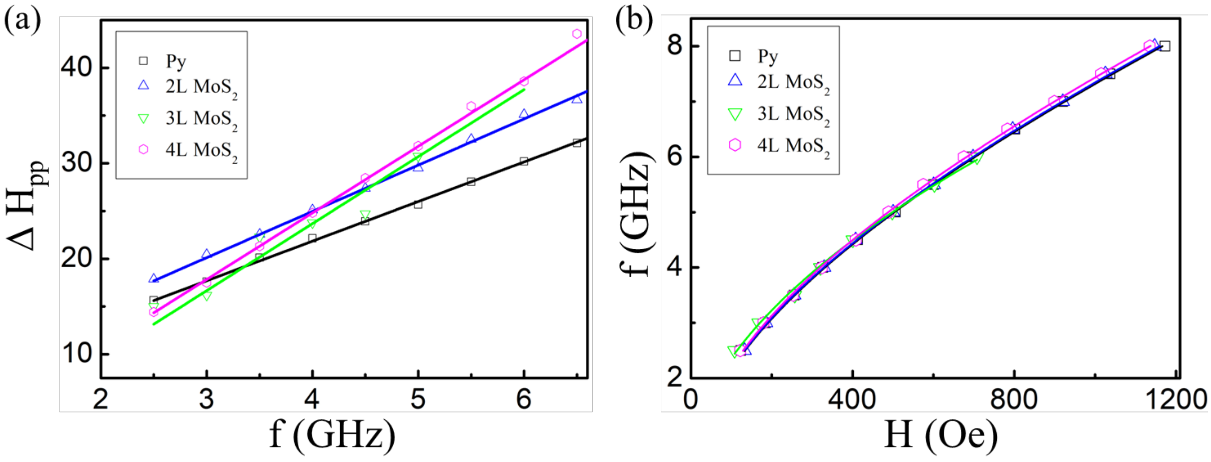

Figure S8. (a) The relationship between the peak-to-peak linewidth of FMR spectra and microwave frequency of 2L-4L MoS_2 samples and pure Py. The solid lines show the linear fits from which the damping factor (α) was derived for each sample. (b) Dependence of the FMR resonance frequency on the external magnetic field for 2L-4L MoS_2 samples and pure Py. The solid lines are the curves fitted according to Kittel formula.

Supplementary Note 7. Numerical fitting procedure of obtained spin-pumping voltage.

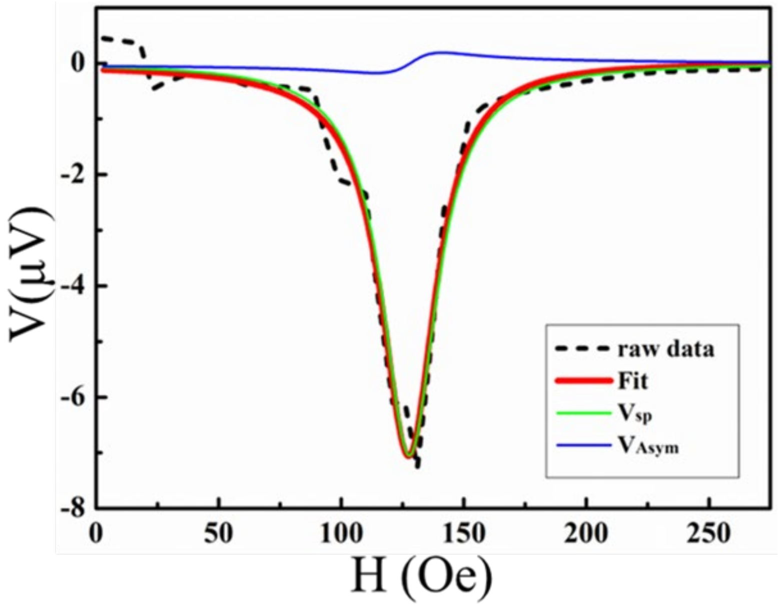

Figure S9. Numerical fitting of measured voltage for Py/MoS_2 sample.
The black, red, green, and blue lines correspond to the original data, the total fit, the fit for symmetric signal (from IEE and ISHE) and the fit for antisymmetric signal (from AMR and AHE), respectively.

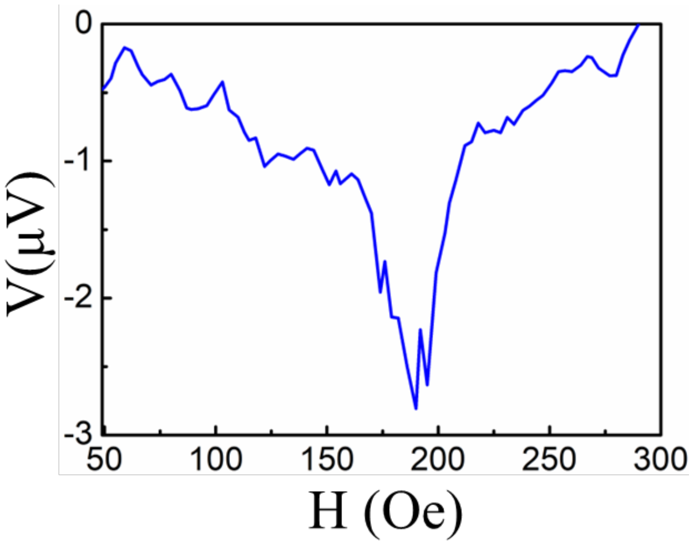

Figure S10. Measured voltage of Py (20 nm) at an excitation frequency of 3 GHz using microwave power 15 mW.

In spin-pumping-induced IEE experiments, the measured voltage may contain spurious signals caused by various effects, such as Nernst effect (NE) and anomalous

Nernst effect (ANE) [5,6], self-induced inverse-spin-Hall effect (ISHE) of Py [7], Seebeck effect [8], anomalous Hall effect (AHE) [7], anisotropic magnetoresistance (AMR) [9], and spin rectification effects (SRE) [10]. Separating these contributions is challenging and various procedures have been proposed based on different symmetry properties [9, 10]. In this study, we use the line-shape analysis method [11-13] to separate the various contributions. The measured spin-pumping voltage V comprises two components, V_{sym} and V_{asym}, as given in eq (1) (in the main text), which are symmetric and antisymmetric with respect to the resonance field (shown in Figure S9). These components can be extracted and estimated using a Lorentzian fit to the data. In the geometry presented in Figures 3a and 4a of the main text, the signal symmetry can easily eliminate the influence of the spin rectification effect on the data [14, 15]. The contributions of IEE and ISHE are symmetric with respect to the resonance field and only affect V_{sym}, whereas the contributions of AMR, SRE, and AHE are antisymmetric with respect to the resonance field [16]. Therefore, we can attribute the symmetric voltage signal to the contributions of ISHE and IEE. To distinguish the contributions of ISHE and IEE, we also measured the voltage induced in pure Py films (grown on sapphire substrates) under the same experimental conditions (i.e., using the same thickness of Py and microwave power). The voltage measured from pure Py film are small (as shown in Figure S10), but thermoelectric signals in Py that induce the NE and ANE may be significant. Our careful studies indicate that the observed symmetric signal mainly arises from the IEE contribution.

Reference:

[1] Samassekou, H.; Alkabsh, A.; Wasala, M.; Eaton, M.; Walber, A.; Walker, A.; Pitkänen, O.; Kordas, K.; Talapatra, S.; Jayasekera, T.; Mazumdar, D. Viable route towards large-area two dimensional MoS2 using magnetron sputtering. *2D Mater.* **2017**, *4*, 021002.

[2] Samassekou, H.; Alkabsh, A.; Stiwinter, K.; Khatri, A.; Mazumdar, D. Atomic-level insights through spectroscopic and transport measurements into the large-area synthesis of MoS_2 thin films. *MRS Communications* **2018**, *8*, 1328-1334.

[3] Jin, W.; Yeh, P.-C.; Zaki, N.; Zhang, D.; Sadowski, J. T.; Al-Mahboob, A.; van der Zande, A. M.; Chenet, D. A.; Dadap, J. I.; Herman, I. P.; Sutter, P.; Hone, J.; Osgood, Jr. R. M. Direct Measurement of the Thickness-Dependent Electronic Band Structure of MoS_2 Using Angle-Resolved Photoemission Spectroscopy. *Phys. Rev. Lett.* **2013**, *111*, 106801.

[4] Lu, S. C.; Leburton, J. P. Electronic structures of defects and magnetic impurities in MoS_2 monolayers. *Nanoscale Res. Lett.* **2014**, *9*, 676.

[5] Huo, Y.; Zeng, F. L.; Zhou, C.; Wu, Y. Z. Spin Pumping and Thermal Effects in Single-Crystalline Fe/Pt Bilayers at the Nonresonant Condition. *Phys. Rev. Applied* **2017***, 8*, 014022.

[6] Yue, D.; Lin, W.; Li, J.; Jin, X.; Chien, C. L. Spin-to-Charge Conversion in Bi Films and Bi/Ag Bilayers. *Phys. Rev. Lett.* **2018**, *121*, 037201.

[7] Tsukahara, A.; Ando, Y.; Kitamura, Y.; Emoto, H.; Shikoh, E.;Delmo, M. P.; Shinjo, T.; Shiraishi, M. Self-Induced Inverse Spin Hall Effect in Permalloy at Room Temperature. *Phys. Rev. B* **2014,** *89*, 235317.

[8] Jiang, Z.; Chang, C.-Z.; Masir, M. R.; Tang, C.; Xu, Y.; Moodera,J. S.; MacDonald, A. H.; Shi, J. Enhanced Spin Seebeck Effect Signal Due to Spin-Momentum Locked Topological Surface States. *Nat. Commun.* **2016**, *7*, 11458.

[9] Azevedo, A.; Vilela-Leão, L. H.; Rodríguez-Suárez, R. L.; Lacerda Santos, A. F.; Rezende, S. M. Spin pumping and anisotropic magnetoresistance voltages in magnetic bilayers: Theory and experiment. *Phys. Rev. B* **2011**, *83*, 144402.

[10] Bai, L.; Hyde, P.; Gui, Y. S.; Hu, C.-M.; Vlaminck, V.; Pearson, J.E.; Bader, S. D.; Hoffmann, A. Universal Method for Separating Spin Pumping from Spin Rectification Voltage of Ferromagnetic Resonance. *Phys. Rev. Lett.* **2013**, *111*, 217602.

[11] Mosendz, O.; Pearson, J. E.; Fradin, F. Y.; Bauer, G. E. W.; Bader, S. D.; Hoffmann, A. Quantifying Spin Hall Angles from Spin Pumping: Experiments and Theory. *Phys. Rev. Lett.* **2010**, 104, 046601.

[12] Mosendz, O.; Vlaminck, V.; Pearson, J. E.; Fradin, F. Y.; Bauer, G. E. W.; Bader, S. D.; Hoffmann, A. Detection and quantification of inverse spin Hall effect from spin

pumping in permalloy/normal metal bilayers. *Phys. Rev. B* **2010**, 82, 214403.
[13] Bai, L.; Feng, Z.; Hyde, P.; Ding, H.F.; Hu, C.-M. Distinguishing spin pumping from spin rectification in a Pt/Py bilayer through angle dependent line shape analysis, *Appl. Phys. Lett.* **2013**, 102, 242402.
[14] Feng, Z.; Hu, J.; Sun, L.; You, B.; Wu, D.; Du, J.; Zhang, W.; Hu, A.; Yang, Y.; Tang, D. M.; Zhang, B. S.; Ding, H. F. Spin Hall Angle Quantification from Spin Pumping and Microwave Photoresistance. *Phys. Rev. B* **2012**, 85, 214423.
[15] Iguchi, R.; Saitoh, E. Measurement of spin pumping voltage separated from extrinsic microwave effects. *J. Phys. Soc. Jpn.* **2017**, 86, 011003.
[16] Martín-Rio, S.; Pomar, A.; Balcells, L.; Bozzo, B.; Frontera, C.; Martínez, B.; Temperature dependence of spin pumping and inverse spin Hall effect in permalloy/Pt bilayers. *J. Magn. Magn. Mater.* **2020**, 500, 166319.
